# Fetal and neonatal outcomes in planned versus unplanned pregnancies in people with spina bifida

**DOI:** 10.1177/1753495X261490378

**Published:** 2026-10-27

**Authors:** Anne Berndl, Georgia Condran, Julia Kfouri, Asia Van Buuren, Paige Church

**Affiliations:** 1 Department of Obstetrics and Gynecology, Division of Maternal-Fetal Medicine, 12366 University of Toronto, Toronto, Canada; 2 Temerty Faculty of Medicine, 12366 University of Toronto, Toronto, Canada; 3 Department of Pediatrics, 8166 University of British Columbia, Vancouver, Canada; 4 Department of Pediatrics, 1862 Boston Children's Hospital, Boston, USA

**Keywords:** Pregnancy, spina bifida, disabled persons, intrauterine growth restriction, congenital abnormality

## Abstract

**Introduction:**

There is limited information on the impact of pregnancy planning on neonatal outcomes in people with spina bifida (SB).

**Methods:**

This study is a sub-group analysis of The Spina Bifida Reproductive Health and Pregnancy Study, an online international cross-sectional survey. Pregnancy, birth and neonatal data were assessed using descriptive and chi-squared analyses.

**Results:**

A total of 209 pregnancies were included: 108 planned and 101 unplanned. The unplanned group included a greater risk of miscarriage/ectopic pregnancy (30%), unplanned fetal loss (34%), intrauterine growth restriction less than 5th percentile (16%) and babies with congenital anomalies. There was no statistically significant difference between unplanned and planned pregnancies in terms of admission to neonatal intensive care unit or risk of preterm birth.

**Conclusion:**

People with SB have rates of unplanned pregnancy similar to the general population. Unplanned pregnancies appear to have increased fetal and neonatal risks. Findings suggest that pregnancy planning is a modifiable factor, which may improve outcomes.

## Introduction

People with disabilities make up approximately 10% of the global population.^1^ Spina bifida (SB) is a congenital neural tube defect characterized by incomplete closure of the spinal column during embryonic development, leading to a range of motor, sensory and urological impairments.^2^ Advances in medical and surgical care have helped improve life expectancy and quality of life in people with SB. A total of 75% of adults with SB now survive into adulthood, allowing many to consider pregnancy.^3^ Although people with disabilities can achieve successful pregnancies, many face unique reproductive health considerations including a lack of physically accessible care environments, mobility concerns, discriminatory attitudes from providers and limited specific reproductive health information for pregnancy planning.^4–6^ As more individuals with disabilities reach reproductive age and choose to bear children, pregnancy in individuals with SB represents a growing area of clinical importance and research.^7–9^

Research to date has shown that people with SB confront a unique spectrum of antenatal risks. A study by Auger et al.^7^ compared 397 pregnant women with SB in Quebec to 1,083,211 women without SB. The study found that babies born to gravidas with SB have an increased risk of adverse neonatal outcomes, including intracranial haemorrhage, birth hypoxia, prolonged hospital stay and oral clefts and abdominal wall defects after adjusting for maternal confounders.^7^ Authors also identified maternal risks including increased hospital intensive care admissions and respiratory morbidity.^7^ An additional study followed 29 pregnancies in 17 women and identified antenatal complications including preterm birth, urinary tract infections and hypertensive disorders in people with SB.^8^ Crane et al. analysed 529 women with spinal cord injury, paralysis or SB with singleton live birth deliveries and found greater risk of urinary tract infection/pyelonephritis, venous thromboembolism, preterm rupture of membranes and caesarean section.^9^ These results demonstrate that there are a growing number of people who are achieving pregnancy; however, people with SB may benefit from enhanced preconception counselling, obstetric monitoring and rehabilitation supports.^7^

Historically, individuals with disabilities have not received equitable access to reproductive and sexual health information to support pregnancy planning.^10,11^ Pregnancy intention has been associated with differences in health behaviour, timing of prenatal care and improved perinatal outcomes in other populations. A recent meta-analysis found that unintended pregnancies in the general population were associated with an increased risk of low-birth-weight babies.^12^ A study of pregnancy planning in people with type 1 diabetes found that unplanned pregnancies were associated with less pre-pregnancy counselling, and MORE small for gestational age infants and admission to the neonatal care unit.^13^ Further research on pregnancy planning in individuals with cerebral palsy and myasthenia gravis has shown that pre-pregnancy consultation leads to more planned pregnancies,^14^ and that planned pregnancies lead to improved pregnancy outcomes.^15^ These findings underscore the critical role of preconception counselling and pregnancy planning in mitigating preventable pregnancy complications.

Minimal information exists on the intersection of SB and reproductive health.^16^ Specifically, the role of pregnancy planning in shaping neonatal outcomes among people with SB remains a limited area of research. As more individuals with disabilities contemplate pregnancy, accessible reproductive care is paramount to supporting pregnancy planning discussions and neonatal health outcomes. This study aims to understand the impact of planned versus unplanned pregnancy on fetal and neonatal outcomes in pregnancies among people with SB to inform targeted preconception counselling and improve reproductive care.

## Methodology

This research is a sub-analysis of the ‘Women with Spina Bifida Reproductive Health and Pregnancy Study’ (WSBRHP), an online international cross-sectional survey examining perinatal outcomes of people with SB. This was a large study that provided an opportunity to gather detailed information on demographics and pregnancy outcomes in a rare population. The study recruitment period was from 2017 to 2019. The current study made use of questions from the WSBRHP regarding pregnancy planning, delivery-related complications and neonatal outcomes. Written informed consent was embedded into the survey. Those who continued to fill out the survey provided consent for their anonymized information to be published in this study. The present study received Research Ethics Board approval through Sunnybrook Health Sciences Centre (REB number: 1142016).

The survey was aimed at self-identified women with SB and was developed using the principles of person-centred research. In line with person-centred principles, the aims and contents of the study were reviewed by people with lived experience to gain their perspective.^17^ Canada Surveys was used as the online platform. The survey was pretested and assessed for feedback by members of Hydrocephalus Canada, followed by testing by two authors to assess for logic within the survey. The online questionnaire was then distributed electronically to international SB organizations to aid in snowball and peer recruitment. Hydrocephalus Canada and the American Spina Bifida Association were involved in this process. Advocacy and support groups were contacted directly to ask if they were comfortable distributing the survey, and they were able to distribute it through various platforms, including newsletters and website links.

The survey was open to anyone; however, screening questions were used to identify those who met criteria. To be included in this sub-analysis, participants needed to speak English, be age 18 and older, be assigned female at birth, have a self-identified diagnosis of SB, experience a pregnancy and self-report whether the pregnancy was planned or unplanned. Individuals who had multiple pregnancies could create separate entries to answer questions related to each pregnancy. Entries were screened for duplications.

Participants responded to optional questions about their pregnancy. Outcomes measured included participant demographics, impacts on their SB and birth and neonatal outcomes, including rates of live and stillbirth, preterm birth and neonatal hospitalizations. Participation rate was calculated as per CHERRIES guidelines. Data was evaluated using descriptive and chi-squared analyses, with a *p* < 0.05 being considered significant.

## Results

The survey was opened 300 times; of these, 253 response sets agreed to participate and met entry criteria, leading to a response rate of 84.7%. Given the open nature of the survey, it is difficult to ascertain how many individuals had access to the survey. The response rate is based on the number of times the survey was opened. Out of 253 responses, 42 did not answer the question ‘how many pregnancies have you had?’. In total, 124 individuals reported a total of 209 pregnancies after duplication removal; 108 were planned and 101 were unplanned (Figure 1). A total of 71 people reported at least one unplanned pregnancy, and the remaining 53 reported only planned pregnancies.

**Figure 1.**
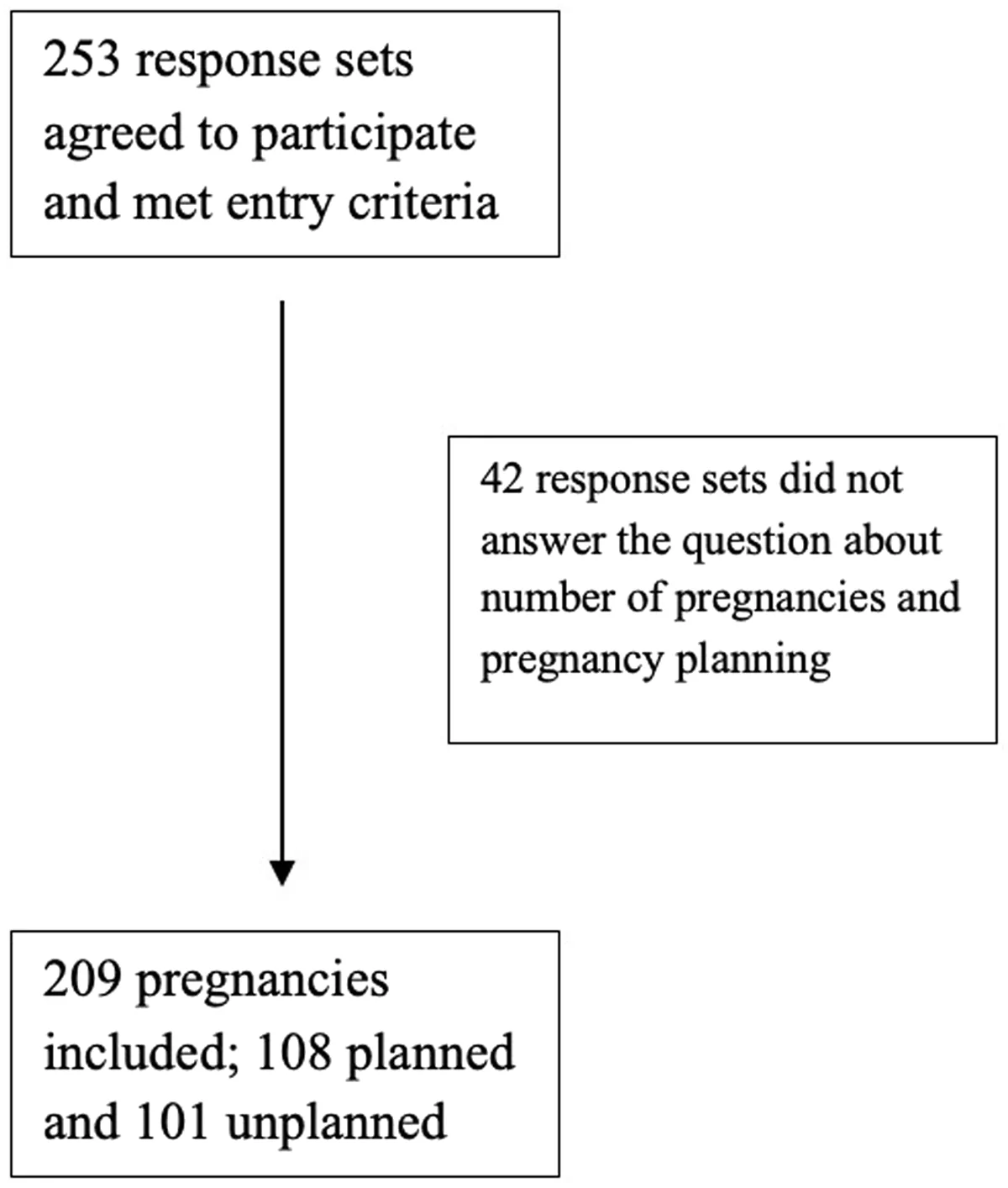
Response set exclusions.

Table 1 illustrates the demographic data of included individuals. The planned group was slightly older and more likely to be married.

**Table 1.** Demographic data of people with spina bifida.

Variable	Planned (*n* = 53) No. (%)	Unplanned (*n* = 71) No. (%)
Average maternal age at first pregnancy (years)	27.7 ± 4.4	24.6 ± 5.6
Average number of pregnancies (including miscarriage, termination of pregnancy, live and stillbirth)	1.90 ± 1.30	2.20 ± 1.19
Country		
United States	28 (52.8)	40 (56.3)
Canada	7 (13.2)	13 (18.3)
Australia	2 (3.8)	5 (7.0)
United Kingdom	2 (3.8)	1 (1.4)
Other	14 (26.4)	12 (16.9)
Marital status		
Single	1 (1.9)	16 (22.5)
Common-law	6 (11.3)	7 (9.8)
Married	38 (71.7)	27 (38.0)
Divorced/separated	6 (11.3)	14 (19.7)
Partnered	1 (1.9)	7 (9.8)
Widowed	1 (1.9)	0 (0.0)
Home service		
No home service	47 (88.7)	58 (81.7)
<20 h per week	5 (9.4)	8 (11.3)
20–60 h per week	0 (0.0)	3 (4.2)
>60 h per week	0 (0.0)	1 (1.4)
Other/blank	1 (1.9)	1 (1.4)
Highest education received		
Less than high school	0 (0.0)	4 (5.6)
High school graduate, diploma or the equivalent	12 (22.6)	13 (18.3)
Some college credit, no degree	7 (13.2)	19 (26.8)
College degree	7 (13.2)	15 (21.1)
Bachelor's degree	16 (30.2)	6 (8.5)
Master's degree	7 (13.2)	3 (4.2)
Professional degree	3 (5.7)	1 (1.4)
Trade, technical, vocational training	1 (1.9)	10 (14.1)
Employment		
Out of work	2 (3.8)	5 (7.0)
Disability pension	10 (18.9)	27 (38.0)
Part-time	9 (17.0)	5 (7.0)
Full-time (>35 h per week)	18 (34.0)	13 (18.3)
Homemaker	8 (15.1)	12 (16.9)
Other	6 (11.3)	9 (12.7)

The impact of SB diagnosis is summarized in Table 2. Each individual was impacted by their SB in some way. Analysis of impact was completed by pregnancy.

**Table 2.** Physiologic and mobility impacts of SB.

Variable	Planned (*n* = 108) No. (%)	Unplanned (*n* = 101) No. (%)
Average maternal age (years)	28.8 ± 4.6	25.6 ± 5.6
Physiologic impacts	88 (81.4)	78 (77.2)
Sensation	88 (81.4)	78 (77.2)
Bladder	86 (79.6)	80 (79.2)
Kidneys	47 (43.5)	48 (47.5)
Bowel	73 (67.6)	76 (75.2)
Seizures	2 (1.9)	1 (1.0)
Shunt	23 (21.3)	46 (45.5)
Sexual dysfunction	11 (10.2)	15 (14.9)
Pain	57 (52.8)	67 (66.3)
Mobility impacts		
None	8 (7.4)	9 (8.9)
Balance and strength only	28 (25.9)	27 (26.7)
Brace	32 (29.6)	20 (19.8)
Walker, cane, walking stick, crutches	27 (25.0)	24 (23.8)
Manual wheelchair	43 (39.8)	47 (46.5)
Power chair	15 (13.9)	13 (12.9)

SB: spina bifida.

There was a total of 209 pregnancies reported. Pregnancy outcomes of planned versus unplanned pregnancies are summarized in Table 3. There were fewer live births and more miscarriages in the unplanned group compared to the planned group. There was the same number of ectopic pregnancies. All stillbirths (4) and terminations (13) were in unplanned pregnancies.

**Table 3.** Outcomes of planned vs unplanned pregnancies in people with spina bifida.

Pregnancy outcome	Planned (*n* = 108)	Unplanned (*n* = 101)	*p*-Value (significant <0.05)
Live birth	95	58	**<0.001**
Stillbirth	0	4	**0**.**037**
Miscarriage	12	25	**0.01**
Ectopic	1	1	0.962
Miscarriage or ectopic	13	26	**0**.**011**
Termination	0	13	**<0.001**

Bold indicate statistically significant.

After excluding terminations, it was found that there was a statistically significant increased risk of miscarriage or ectopic in the unplanned group (26/88; 30%) compared to the planned group (13/108; 12%) (*p*-value = 0.011039). The unplanned group also had a higher risk of unplanned fetal loss, including stillbirth (30/88; 34% compared to 13/108; 12%) (*p*-value = 0.001593).

Fetal and neonatal outcomes in planned versus unplanned pregnancies resulting in live birth or stillbirth (defined as pregnancy loss greater than 20 weeks’ gestation) are summarized in Table 4. The unplanned group was associated with significantly more intrauterine growth restriction (IUGR) less than 5th percentile (10/62; 16% vs 6/94; 6%) (*p*-value = 0.04959) and congenital anomalies (9/48; 19% vs 3/85; 4%) (*p*-value = 0.008220).

**Table 4.** Fetal and neonatal outcomes among people with spina bifida.

Pregnancy outcome	Planned	Unplanned	*p*-Value (significant <0.05)
Congenital anomalies	3/85	9/48	**0** **.** **008**
IUGR <10th percentile	14/94	15/62	0.144
IUGR <5th percentile	6/94	10/62	**<0.05**
IUGR <3rd percentile	4/94	7/62	0.093
NICU admission	17/85	11/52	0.692
Preterm birth	24/94	14/62	0.674

IUGR: intrauterine growth restriction; NICU: neonatal intensive care unit.

Bold indicate statistically significant.

Neonatal intensive care unit (NICU) calculations excluded stillbirth. In total, 85 of planned live births and 48 of unplanned live births provided data on NICU admission. There was no significant difference in NICU admission of liveborn babies in planned compared to unplanned pregnancies (11/48; 23% vs 17/68; 25%) (*p*-value = 0.691921). Further, there was no difference when analysing NICU with stillbirth, and no NICU or stillbirth, in planned versus unplanned pregnancies (*p*-value = 0.235006).

There was no increased risk of preterm birth when comparing planned to unplanned pregnancies (14/62; 23% vs 24/94; 26%) (*p*-value = 0.67431).

The unplanned group reported nine liveborn babies with congenital anomalies compared to three in the planned group (Table 5). Anomalies in the planned group included primary pulmonary hypertension, SB with hydrocephalus and SB. Conversely, congenital anomalies in the unplanned group included club foot, cardiac abnormality, vascular ring, tongue-tie and SB.

**Table 5.** Congenital anomalies of newborns in planned vs unplanned pregnancies in people with spina bifida.

Pregnancy group	Congenital anomaly
Planned (*n* = 85)	Primary pulmonary hypertension Spina bifida and hydrocephalus Spina bifida
Unplanned (*n* = 48)	Club foot Cardiac abnormality Cardiac abnormality Vascular ring Vascular ring with tongue-tie Vascular ring with tongue-tie Tongue tie Tongue tie Spina bifida

## Discussion

People with SB appear to have similar rates of unplanned pregnancies to the general population.^18^ In this study, we found that unplanned pregnancies were associated with increased fetal and neonatal risks, including significantly increased rates of miscarriage, stillbirth, IUGR less than 5th percentile and congenital abnormalities, when compared to planned pregnancies in individuals with SB. However, no significant differences between groups were observed in rates of preterm birth or NICU admission. These findings suggest that pregnancy intention may play a meaningful role in modulating fetal and neonatal outcomes among gravidas with SB.

Historically, conversations about sexual and reproductive health among people with disabilities were limited by healthcare provider misperceptions about sexual activity and desire for conception in the group.^6,11,19^ Pregnancy planning has been identified as a modifiable risk factor in other conditions, including myasthenia gravis, lupus and cerebral palsy.^14,15,20^ A study by Wotherspoon et al.^13^ found that unplanned pregnancies among people with type 1 diabetes mellitus were associated with increased rates of small for gestational age, NICU admission and prolonged hospitalization. Pregnancy planning allows for improved preconception counselling and medical optimization prior to gestation, which helps improve perinatal outcomes.^13^ Specific components of pregnancy planning that may improve outcomes include starting high-dose folic acid prior to conception,^21^ optimizing medication, optimizing bladder concerns,^22^ optimizing physical fitness,^23^ treatment of anaemia,^24^ establishing mental health support^25^ and allowing the opportunity to plan financially and optimize support systems.

People in the unplanned and planned groups had similar baseline SB and demographic characteristics. Individuals in the planned group were older, more likely to have post-graduate education experience, and more likely to be married. Current research suggests that increased age during pregnancy may be associated with poor outcomes, including gestational diabetes, preeclampsia and preterm birth.^26^ However, it is possible that pregnancy planning led to decreased obstetric risks, despite advanced maternal age, suggesting that pregnancy planning has significant benefits in relation to obstetric, neonatal and post-partum outcomes. This information is limited by the retrospective nature and sample size of the study.

Of note, there appear to be more people with shunts in the unplanned group. There is currently limited available information on the outcomes of people with shunts in pregnancy.^27^ This highlights an important gap in knowledge. Future research should explore if people with hydrocephalus are predisposed to unplanned pregnancies (e.g. due to limited access to reproductive care or contraception) or if shunts and certain hydrocephalus characteristics are associated with worse pregnancy outcomes.

The results of the current study are aligned with past research, which shows that pregnancies among people with SB are associated with increased rates of infant complications at delivery.^7^ Specifically, the unplanned group was associated with increased rates of IUGR less than 5th percentile, which is associated with significant neonatal morbidity. The additional risks associated with unplanned conception in our cohort likely reflect the absence of preconception optimization, including folic acid supplementation and medication review to optimize seizure medications to reduce the risk of teratogenic effects.^28^ Preconception physiologic assessment may also help to reduce the risk of adverse pregnancy outcomes, including engaging in smoking cessation and improving physical fitness, which has been positively correlated with neonatal weight.^29^

Pregnancies in the planned group had similar rates of congenital anomalies to the general population, whereas the unplanned group demonstrated significantly increased rates of congenital anomalies.^30^ It has been shown that a history of neural tube defect, including SB, is a significant risk factor for the development of similar congenital anomalies.^31^ The present study found an increased risk of SB and other fetal malformations. Interestingly, we found no difference in preterm birth or NICU admission between planned and unplanned pregnancy. This diverges from prior studies in the general population where unplanned pregnancies were associated with preterm birth and increased neonatal morbidity.^32^

There are limitations inherent to survey research, including reliance on self-reported data and retrospective nature. The primary limitation was the small sample size, which limits generalizability as the sample may not be fully representative of the spectrum of SB. This also limits the ability to perform multivariate analysis. In addition, questions were not mandatory, and analyses were completed per pregnancy (as some individuals experienced more than one pregnancy) on questions with responses. We did not perform sensitivity analysis on only first pregnancies as it was felt that the sample size would be too small given the rareness of the condition. This is a possible limitation given that later pregnancies may have fewer complications than the index pregnancy. Further, the study is subject to sources of bias inherent to survey research, which may limit the generalizability of findings and should be considered when interpreting results. For example, sample bias may have occurred as participants were recruited from organizations, potentially resulting in a sample that is more engaged with healthcare or support services. Further, response bias is possible, as individuals with particular interest in reproductive health, or those with positive or negative pregnancy experiences may have been more likely to participate. Non-response bias cannot be excluded. Finally, recall bias is an important consideration, particularly for individuals reporting on pregnancies that occurred many years prior to survey completion. People who responded to the survey had pregnancies in different time periods, which could lead to variability in responses. The strengths of this study lie in the inclusion of detailed demographic data and specific questions related to pregnancy planning, which may help to inform approaches to preconception counselling for multi-disciplinary providers.

These results support the notion that a growing number of people with SB are achieving pregnancy, but pregnancy outcomes remain under-studied. People with SB appear to have a high risk of fetal and neonatal morbidity, which may be mitigated by pregnancy planning and preconception counselling with multi-disciplinary teams. This study suggests that pregnancy planning may be an important modifiable risk factor for people with SB. There is an opportunity for enhanced obstetric management in this group to help reduce preventable fetal complications and congenital abnormalities.

## Conclusion

People with SB have rates of unplanned pregnancy similar to the general population. Unplanned pregnancies in people with SB appear to be associated with increased fetal and neonatal risks, including miscarriage, stillbirth, IUGR and congenital abnormalities. This is the first study assessing the fetal and neonatal outcomes of planned versus unplanned pregnancy in people with SB. Findings suggest that pregnancy planning is a modifiable risk factor that can lead to improved outcomes in people with SB, emphasizing the critical role of preconception counselling, early antenatal assessment and multi-disciplinary care in this group.
